# Evaluation of Objective Signs and Subjective Symptoms of Dry Eye Disease in Patients with Inflammatory Bowel Disease

**DOI:** 10.1155/2019/8310583

**Published:** 2019-01-08

**Authors:** Zsolt Barta, Levente Czompa, Aniko Rentka, Eva Zold, Judit Remenyik, Attila Biro, Rudolf Gesztelyi, Judit Zsuga, Peter Szodoray, Adam Kemeny-Beke

**Affiliations:** ^1^Department of Gastroenterology, Institute of Medicine, Faculty of Medicine, University of Debrecen, Nagyerdei krt. 98, 4032 Debrecen, Hungary; ^2^Department of Infectology, Faculty of Medicine, University of Debrecen, Bartok B. u. 2-26, 4031 Debrecen, Hungary; ^3^Division of Oral and Maxillofacial Surgery, Faculty of Dentistry, University of Debrecen, Nagyerdei krt. 98, 4032 Debrecen, Hungary; ^4^Department of Ophthalmology, Faculty of Medicine, University of Debrecen, Nagyerdei krt. 98, 4032 Debrecen, Hungary; ^5^Department of Clinical Immunology, Institute of Medicine, Faculty of Medicine, University of Debrecen, Moricz Zsigmond krt. 22, 4032 Debrecen, Hungary; ^6^Institute of Food Technology, Faculty of Agricultural and Food Sciences and Environmental Management, University of Debrecen, Boszormenyi ut 138, 4032 Debrecen, Hungary; ^7^Department of Pharmacology and Pharmacotherapy, Faculty of Medicine, University of Debrecen, Nagyerdei krt. 98, 4032 Debrecen, Hungary; ^8^Department of Health Systems Management and Quality Management in Health Care, Faculty of Public Health, University of Debrecen, Nagyerdei krt. 98, 4032 Debrecen, Hungary; ^9^Institute of Immunology, Oslo University Hospital, Rikshospitalet, Sognsvannsveien 20, 0372 Oslo, Norway

## Abstract

**Aim:**

To evaluate tear film parameters and relationship of objective clinical signs and subjective symptoms of dry eye disease (DED) in inflammatory bowel disease (IBD) subgroups.

**Methods:**

39 patients with Crohn's disease (CD), 26 patients with ulcerative colitis (UC), and 39 control persons with no ocular symptoms or surface disorders were included in this prospective, case-control, and cross-sectional study. The ocular surface disease index (OSDI) questionnaire was applied to evaluate dry eye symptoms, and objective tests of DED were performed on both eyes of each subject.

**Results:**

The average of OSDI scores was 30.59 (±16.68) in CD patients, 24.67 (±23.48) in UC patients, and 11.19 (±5.8) in controls. Except for tear film breakup time (tBUT) and Schirmer-I values other objective parameters were better in UC patients, than in CD patients. CD patients rather than UC patients tend to develop DED. This was associated with immunosuppressant and TNF-*α* inhibitor use.

**Conclusions:**

Clinicians must be aware of the spectrum of DED involvement in IBD and suggest using artificial tears in order to decrease severity of ocular complications.

## 1. Introduction

Inflammatory bowel disease (IBD) including Crohn's disease (CD) and ulcerative colitis (UC) is a group of chronic inflammatory disorders principally affecting the intestines, but, being a systemic immune-mediated illness, it is not limited to the gastrointestinal (GI) tract [1–3]. It falls under the spectrum of global diseases as both its incidence and prevalence are increasing with an uneven occurrence in different regions all over the world [4]. The etiopathogenesis of IBD is complex and not well known, probably multifactorial. It is thought to be the result of a dysregulated immune response to gut microbiota in a genetically predisposed host, arising at a confluence of genetic and external environmental influences [5]. Extraintestinal symptoms of IBD can be split into two subgroups: extraintestinal manifestations (EIM) and extraintestinal complications (EC). EIMs are primary systemic affections by the disease itself, while ECs are secondary lesions caused by malnutrition, chronic inflammation, or side effects of therapy. EIMs are reported to occur from 6 up to 40% of patients diagnosed with IBD, affecting the musculoskeletal and mucocutaneous systems, the eyes, and the hepatobiliary tract, and can be more debilitating than the fundamental IBD itself. The pathophysiology of ocular EIMs (O-EIM) of IBD has not been clearly explored, but the immunologic nature of the underlying disease should be of significant importance. Antibody productions or antigen-antibody complexes can pass through the mucosal intestinal epithelium damaged by inflammation and they can cross the blood-retina barrier and generate inflammation of the eye. In addition microbial pathogens from gut may contribute to altered immune mechanisms and as a consequence O-EIM can develop. Yet another interpretation is that genetic factors or local coexistent damage factors could modulate the display of potential antigens and autoimmune flairs can evolve [6–8]. No significant association of age and the occurrence of O-EIMs in IBD was found by a prospective clinical study of a large population of IBD patients performed by Yilmaz et al. [9].

Within ECs the incidence of ocular complications (OCs) features 2%-6%, occurring more frequently in CD than in UC [10–13].

In a review article Mady and coworkers summarized OCs and their treatments in IBD. They state that ocular manifestations do not always coincide with active intestinal flare. They draw the attention to the importance of early diagnosis of IBD since ocular involvement can antedate intestinal disease therefore if the diagnosis of IBD is made before the GI tract is sorely affected many long term consequences might be possible to avoid or at least delay [14]. They may be diagnosed before, concurrently, or after the diagnosis of IBD.

OCs are distinguished by Knox et al. as primary, secondary, and coincidental. Primary OCs, e.g., episcleritis, scleritis, keratopathy, and uveitis, are temporarily related to the activity of IBD and cured by conservative treatment or surgical intervention of the intestinal inflammation. Secondary OCs are due to primary OCs. Examples include posterior subcapsular cataract formation as a result of corticosteroid administration, scleromalacia due to scleritis, episcleritis, or underlying vasculitis, and night blindness, by reason of hypovitaminosis A following a low-vegetable diet or gut resection. Coincidental complications are ocular disorders that do not correlate with IBD. This group contains, e.g., conjunctivitis, photophobia, or subconjunctival hemorrhage [15].

DED is considered to be an uncharacteristic OC of IBD, and although DED can dramatically reduce quality of life (QOL) in the affected population [16], its importance is underestimated, and a correlation between IBD and DED has rarely been investigated (especially not compared UC and CD subgroups).

IBD affects multiple organs including the eyes, but although there is an unmet need for research on EIMs in IBD in general, which specifically holds true regarding ocular manifestations, there are only scant publications available concerning ophthalmological involvements in the course of the disease, and tear deficiency alterations have rarely been reported. Felekis et al. analyzed 60 IBD (37 UC and 23 CD) patients focusing on ophthalmologic manifestations and tBUT and Schirmer tests and rose-bengal corneal staining was used to investigate DED. They found that 50% of patients suffered from DED, by far the most frequent ocular manifestation of IBD, which was considered as a secondary OC [17]. Cury and Moss introduced the data of 48 patients with CD, 40 patients with UC, and 24 controls. They found that eye symptoms were common in patients with IBD, and DED was present in 44% of patients. They also revealed a strong correlation between 5-ASA use especially in doses >3 g per day and DED [18]. On the contrary Cloché et al. did not find any relationship between DED and IBD-related medications in their prospective observational study. They also qualified DED as a secondary OC of IBD [19]. Lee et al. examined 36 patients with CD and 25 with UC and stated that the most common coincidental O-EIM was DED supported by a prevalence of 57% compared to rate of 21.3% in the control group [20]. Li and colleagues stated 2% O-EIMs including DED in IBD patients in the Chinese population, which is fairly lower than the rates reported in the studies of European and American countries [21].

The primary aim of our study was to evaluate various tear film parameters and subjective symptoms of DED in patients with IBD (UC or CD) and compare them with those of healthy controls, while the secondary aim was to appraise associations among objective values and clinical variables.

## 2. Patients and Methods

### 2.1. Features of Patients

Unselected and consecutive patients of the Department of Gastroenterology were invited to participate. The diagnosis and phenotype of CD and UC were established by an experienced gastroenterologist using clinical, imaging, endoscopic, and histopathological criteria in accordance with the Montreal Classification [22]. None of the patients fulfilled the diagnostic criteria for secondary Sjögren's syndrome (SS) or developed secondary SS during the one-year follow-up period. All patients had at least 1 colonoscopy done throughout the duration of the disease, and at the time of investigations the underlying IBD was inactive. Age, disease duration of IBD patients, and medications used within 6 months prior to evaluation and concurrent medications were recorded.

### 2.2. Features of Controls

An age- and gender-matched population for control was also enrolled in the study during the same period. Controls came into the Department of Ophthalmology for routine ophthalmological examination and presented without any corneal disease or tearing alteration and had no history of digestive symptoms or any systemic or immune-related diseases. They had minor refractive errors (±1.0 diopter), consequently slight reading or distance vision disabilities, and they were invited to voluntarily participate in the study.

### 2.3. Exclusion Criteria

Patients and members of the control group were not allowed to enter the study if they had taken any eye drops in the two weeks prior to tear sampling. Other exclusion criteria were preexisting ocular disease such as glaucoma or uveitis, previous ocular surgery, abnormal eyelid position and closure, disorders of the nasolacrimal drainage system, and contact lens wearing. Patients and members of the control group were allowed to enter the study if they had not taken any eye drops for two weeks prior to tear sampling. Other exclusion criteria were preexisting ocular disease such as glaucoma or uveitis, previous ocular surgery, abnormal eyelid position and closure, disorders of the nasolacrimal drainage system, and contact lens wearing.

Patients underwent a comprehensive ophthalmological evaluation, including best corrected visual acuity (BCVA) using a Snellen chart, intraocular pressure (IOP) measurement, and broad beam examination of the slit lamp to determine the condition of the ocular surface and surrounding tissues, to observe tear film, corneal impairments, conjunctival changes, and eyelids, and fundoscopy.

The study protocol was approved by the local ethics committee and was in full compliance with Good Clinical Practices, and the Declaration of Helsinki (1996). By signing a written informed consent all patients agreed to have the study results regarding any side effects as well as possible risks and benefits of the study published.

#### 2.3.1. OSDI

To assess subjective symptoms of DED Ocular Surface Disease Index (OSDI) questionnaire was applied since it has been highly recommended by the US Food and Drug Administration (FDA) for use in clinical studies. All questions focus on the last one-week time interval before test administration. All patients and controls were asked by a trained interviewer. This questionnaire is subdivided into three subscales, and the final score is determined by an algorithm. The overall OSDI score characterizes the ocular surface as normal (from 0 to 12 points) or as having a mild (from 13 to 22 points), moderate (from 23 to 32 points), or severe (above 33 points) disease [23, 24].

Then the following five separate examinations were performed. During investigations special attention was paid to the circumstances: all examinations were carried out on sequential days, and invariant light, temperature, and humidity were ensured in order to exclude any ocular surface stress.

#### 2.3.2. Tear Film Stability: Tear Breakup Time (tBUT)

To assess tear film stability a fluorescein-impregnated strip wetted with a drop of unpreserved, sterile saline solution 0.9% was touched to the lower bulbar conjunctiva. After some blinking the tear film was examined under cobalt blue light. The interval between the last blink and the occurrence of the first dry spot was considered as the tBUT. Three tBUT measurements for both eyes of all subjects were performed and the average of them was determined as mean value.

#### 2.3.3. Tear Quantity Measurement: Schirmer-I Test (ST_I_)

To determine tear quantity Unanesthetized Schirmer test, the ST_I_ was carried out. Standardized Schirmer strips (Alcon Laboratory, Fort Worth, Texas, USA) were inserted in the lower eyelid pouch of both eyes with special attention to avoid touching the cornea. Patients and controls were instructed to softly close their eyes for 5 minutes; then the strips were taken out and the amount of moisture was measured. The main value was calculated as the average of both ST_I_ values.

#### 2.3.4. Vital Staining: Lissamine Green (LG) Score

For vital staining an LG impregnated paper strip (HUB Pharmaceuticals, Rancho Cucamonga, CA, USA) formerly dampened with a drop of unpreserved, sterile saline solution 0.9% from a single-dose ampule was stroked to the lower bulbar conjunctiva. After a few blinks the ocular surface was evaluated under slit lamp in the manner suggested by Foulks [25]. The final LG scores were determined as per Bron's schema (Oxford Grading Charts): grade 0 was considered when bulbar conjunctiva comprised 0 to 9 dots, grade 1 with 10 to 32 dots, grade 2 with 33 to 100 dots, and grade 3 beyond 100 dots.

#### 2.3.5. Lid Parallel Conjunctival Fold (LIPCOF)

In order to evaluate the severity of dry eye lid parallel conjunctival fold (LIPCOF) determination was used since folds in the lateral, lower quadrant of the bulbar conjunctiva, parallel to the lower lid margin are significantly related to dry eye [26, 27]. During LIPCOF test subjects were asked to blink a few times and then fix straight-ahead. Horizontal conjunctival folds at the territory from the middle to the temporal third of the lower eyelid were investigated under a slit lamp starting with low level of illumination and then gradually intensifying the level. In the course of evaluation the size of the conjunctival folds and also the height of the normal tear meniscus were taken into account. Degree 0 was recorded when in case of no persistent fold was present degree 1 if a single small fold in the primary eye position appeared smaller than the normal tear film meniscus, degree 2 if multiple folds were up to the height of normal tear meniscus, and degree 3 if multiple folds were higher than the normal tear meniscus [28].

### 2.4. Statistical Analyses

Data of patients and controls were compared by one-way ANOVA after verifying the Gaussian distribution of data with D'Agostino & Pearson and Shapiro-Wilk normality tests. Mann-Whitney* U* test was used in case of nonparametric distribution. Correlation coefficients between variables were calculated with the Pearson or Spearman's methods (*r*). Data are presented as mean (± SD). P values less than 0.05 were considered statistically significant. For the statistical analysis, IBM SPSS 24 statistical software (IBM Corp., Armonk, New York, USA) was used.

## 3. Results

Thirty-nine patients with histologically confirmed CD (21 female and 18 male), mean (± SD) age 42.26 (±12.36) years, and 26 patients (7 female and 19 male), mean age 46.38 (±12.75) years with histologically confirmed UC were recruited into our study; all were enrolled from the outpatient clinic of the Department of Gastroenterology. The average disease duration was 12.74 (±6.63) years for CD patients, while it was 11.27 (±7.34) years for UC patients. We enrolled 39 gender- and age-matched volunteers (24 females and 15 males) mean age 48.51 (±15.92) years as healthy controls. All study subjects were of Caucasian origin. There was no significant difference between demographic data of male or female patients and their healthy counterparts in any examined parameter.

**Figure 1:**
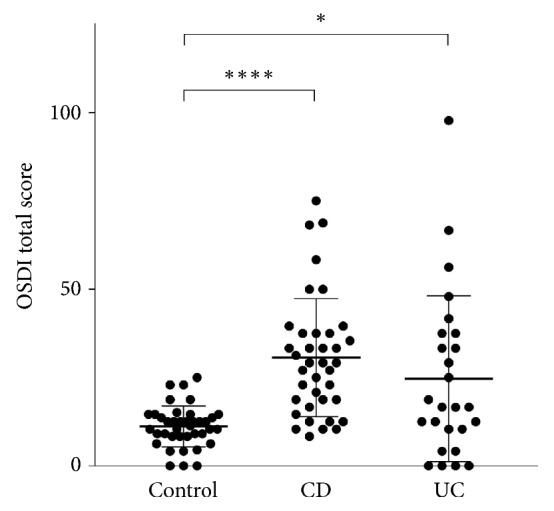
OSDI total scores of controls, CD, and UC patients. OSDI: ocular surface disease index; CD: Crohn's disease; UC: ulcerative colitis.

**Figure 2:**
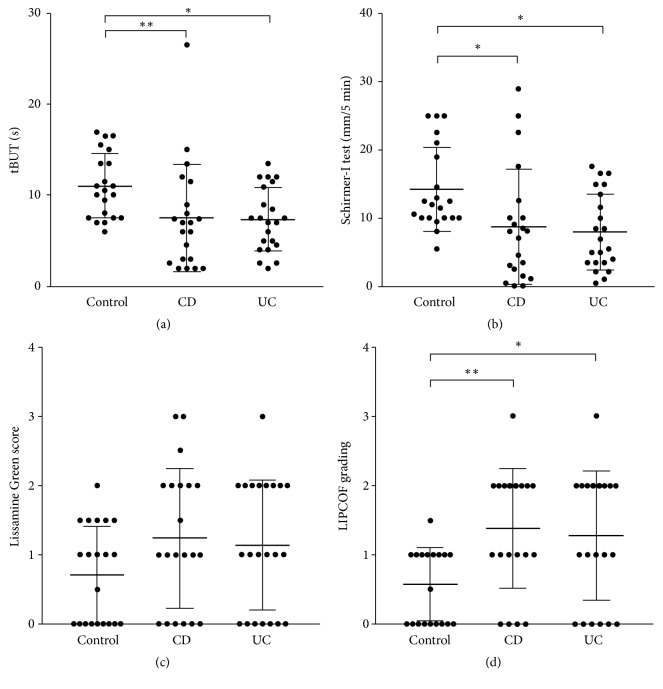
tBUT (s), Schirmer-I (mm/5 min) values, Lissamine green score, and LIPCOF scores in tears of controls, CD, and UC patients. (a) tBUT (s); (b) Schirmer-I test (mm/5 min); (c) Lissamine green scores; (d) LIPCOF grading values of controls, CD, and UC patients. tBUT: tear breakup time; LIPCOF: lid parallel conjunctival folds.

The average OSDI scores were 10.88 (±5.54) in controls, 30.59 (±16.68) in CD patients (p=0.0001), and 24.67 (±23.48) in UC patients (p=0.0550) (Figure 1). CD patients had more impaired objective parameters of DED than UC patients since except for LG scores there were significant differences between measurements of CD patients and healthy controls, but data of UC patients were nearer to those of the control group. Data are represented in Table 1 and Figure 2. Medications of patients used within 6 months prior to evaluation are shown in Table 2.

**Figure 3:**
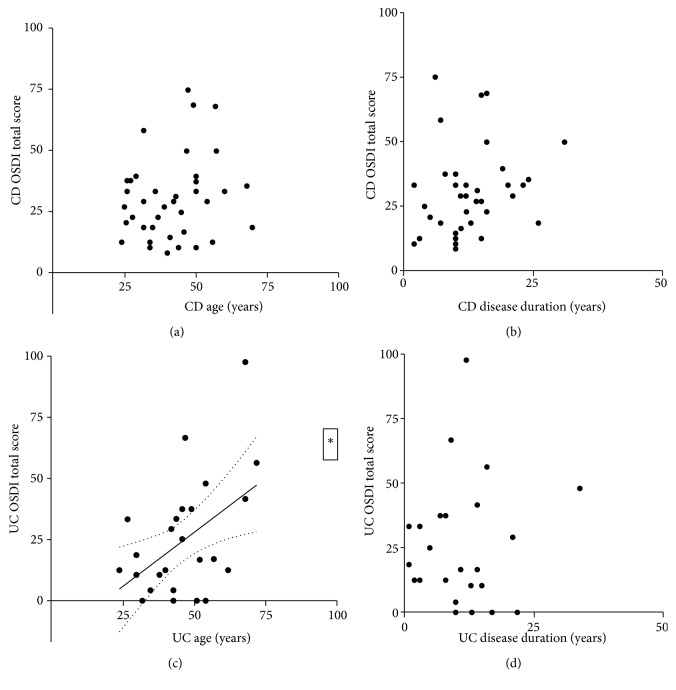
Correlation between OSDI total scores and age as well as disease duration in CD and UC patients. The continuous lines show the fitted curves, while the dotted lines represent the 95% confidence bands (obtained with linear regression in cases when there was a significant correlation between the data sets). (a) CD OSDI total score and CD age; (b) CD OSDI total score and CD disease duration; (c) UC OSDI total score and UC age; (d) UC OSDI total score and UC disease duration. OSDI: ocular surface disease index; CD: Crohn's disease; UC: ulcerative colitis.

In general, weak associations were found as a result of correlation analysis between objective signs and age and disease duration of CD and UC patients. Associations between OSDI scores as a subjective parameter and patients' age and disease duration are represented in Figure 3. A significant positive correlation was only found between OSDI and age of UC patients (r = 0.5302, p = 0.01). Association between OSDI scores and objective signs of DED in controls as well as in patients is shown in Table 3. Except for tear production, associations between OSDI scores and objective test results have been confirmed in the control group. Except for LG score a considerable discrepancy was disclosed between objective clinical parameters and OSDI scores in CD patients, while only a slight difference was revealed between these values in UC patients (Table 3.)

## 4. Discussion

Since our study deals with DED it is interesting to examine risk factors that lead to this condition. Evaluating the data of a large cohort the prevalence of DED is increasing with age; in patients older than 80 years it reached 19%. Also history of arthritis, thyroid disease, gout, smoking, caffeine and multivitamin use, and diabetes are independent of each other and significantly associated with DED [29]. Since a couple of them have immunological aspects, and IBD also has an immunological feature in etiology, IBD patients are prone to develop DED, mostly in old age.

This is the first study investigating subjective signs and objective symptoms of DED in patients with IBD, assessing the association of patients' age, disease duration, treatment, and signs and symptoms. Our data affirm the finding that CD patients rather than UCs tend to develop DED signs, because in regard to ocular tear film characteristics they have more impaired parameters than UC patients.

In our study, however, OSDI values (representing subjective symptoms) of healthy controls showed significant correlation with all objective parameters of tear production and tear film stability. At the same time, the correlation between subjective and objective parameters was almost the same for UC patients. In contrast, this correlation was considerably disturbed in CD patients: only LG scores correlated significantly with OSDI values (Table 3). Both CD and UC are diseases that show no correlation with age or disease duration in regard to DED. The association between objective signs and subjective symptoms present in the healthy control group is much poorer in CD than in UC. Regarding medications 5-aminosalicylates (5-ASA) products were prescribed for the majority of patients both in the CD group and in the UC group: 95% and 92%, respectively. There were no significant differences between 5-ASA and systemic and topical steroid medications, but significant differences were confirmed related to immunosuppressants and TNF-*α* inhibitors (p=0.0430 and 0.0143, respectively).

Clinicians must be aware of the diverse spectrum of the OCs associated with IBD and accordingly include evaluation of the eye and moreover might tell IBD patients to use artificial tear drops and ointments. Considering that IBD patients above 40 years of age are more likely to have OCs than those under 40 [7], sight-threatening complications may be avoided by early diagnosis and treatment of DED. Rarely, eye manifestations can anticipate the IBD diagnosis [21], and early diagnosis of IBD has even importance in corneal refractive surgery since unstable or uncontrolled status of immune-mediated diseases is regarded as an absolute contraindication for keratorefractive surgeries [30–32].

IBD patients, suffering from a relatively rare disease, spend most of their time among presumably healthy peers, who are in a relatively better eye condition; therefore IBD patients are liable to forget their treatment against DED. CD patients have pronounced subjective feelings about dry eye sensation as compared to UC patients, who are not much confronted with their imperfections. Interestingly enough, however, all objective parameters of tears were altered for both CD and UC patients. Therefore UC patients' attention should be drawn for applying therapy against DED in order to maintain ocular surface health.

Our study found significant differences regarding tear film characteristics between CD patients and healthy controls, but these data of UC patients were closer to normal values than those of the CD patients except ST_I_ and tBUT values. These findings confirm the fact that although in UC the values of tear quantification are quite similar to those of normal controls, the quality is diminished, which should be explained by the altered composition of tears. In our study a significant difference was demonstrated in the use of immunosuppressants and TNF-*α* inhibitors between the two patient groups. Reviewing the literature ocular manifestations do not feature as side effects of immunosuppressant use but they can modulate the immune system and trophic functions of the main and the accessory lacrimal glands that may result in DED [33]. As for the use of TNF-*α* inhibitors conflicting results have been reported about TNF-*α* blocking agents' tear production [34, 35]. IBD, considered to be a chronic inflammatory disorder, can modify the immune system and activity of the main and the accessory lacrimal glands that produce tears.

Further investigations are required to study the effect of CD and UC on tear quantity and quality and effect of medications on tear parameters. Since the main limitations of the study could be the relatively small sample size, and lack of information about comorbidities, we actually intend to increase the number of enrolled patients and controls and incorporate their data.

Ocular manifestations of IBD are unjustly underrepresented in the literature, although they signify a substantive problem. While diagnosing IBD patients they should be referred to as an ophthalmologist in order to verify/exclude the presence of DED, a possible EIM of the disease.

## Figures and Tables

**Table 1:** Characteristics and comparison of control persons with CD and UC patients presented as mean±SD. CD: Crohn's disease; UC: ulcerative colitis; tBUT: tear breakup time; LIPCOF: lid parallel conjunctival folds; OSDI: ocular surface disease index; Co: control.

	**Control**	**CD**	**UC**	**p: Co *vs* CD**
				**p: Co *vs* UC**
Age (years)	48.51 ± 15.92	42.26 ± 12.36	46.38 ± 2.75	0.2015
				0.6071

Disease duration (years)	-	13.9 ± 7.98	12 ± 7.52	(CD vs UC)
				0.4249

tBUT (s)	11.23 ± 2.98	7.43 ± 5.16	7.52 ± 3.59	**0.0075**
				**0.0214**

Schirmer I test (mm/5 min)	13.19 ± 4.86	9.11 ± 7.58	8.52 ± 6.13	**0.0328**
				**0.0112**

Lissamine Green score	0.59 ± 0.63	1.14 ± 0.91	1.12 ± 0.99	**0.0282**
				0.2758

LIPCOF grading	0.51 ± 0.52	1.31 ± 0.82	1.19 ± 0.94	**0.0065**
				**0.0219**

OSDI total score	10.88 ± 5.54	30.59 ± 16.68	24.67 ± 3.48	**0.0001**
				0.0550

**Table 2:** Medications of patients. CD: Crohn's disease; UC: ulcerative colitis; 5-ASA: 5-aminosalicylates.

**Medications**	**CD (**%**)**	**UC (**%**)**	**P value**
Systemic steroids	22 (56)	18 (70)	>0.9999
Topical steroids	18 (46)	16 (62)	>0.9999
5-ASA	37 (95)	24 (92)	0.4035
Immunosuppressants	19 (49)	12 (46)	**0.0430**
TNF-*α* inhibitors	15 (38)	6 (23)	**0.0143**

**Table 3:** Correlation of subjective symptoms (OSDI total score) with objective signs characterizing dry eye disease (tBUT, Schirmer test, Lissamine green score and LIPCOF grading) in control persons (Control panel), Crohn's disease patients (CD panel), and ulcerative colitis patients (UC panel). CI: confidence interval; tBUT: tear breakup time; LIPCOF: lid parallel conjunctival folds; OSDI: ocular surface disease index.

**Control**	OSDI vs. tBUT (s)	OSDI vs. Schirmer test (mm/5 min)	OSDI vs. Lissamine Green score	OSDI vs. LIPCOF

r (Pearson)	-0.3344	-0.3532	0.3658	0.5076

95% CI	-0.5909 to -0.01649	-0.6046 to -0.03784	0.05223 to 0.6137	0.2243 to 0.7118

p value	**0.0402**	**0.0296**	**0.0239**	**0.0011**
	**∗**	**∗**	**∗**	**∗** **∗**

**CD**	OSDI vs. tBUT (s)	OSDI vs. Schirmer test (mm/5 min)	OSDI vs. Lissamine Green score	OSDI vs. LIPCOF

r (Pearson)	-0.09736	-0.05682	0.3399	0.1243

95% CI	-0.4127 to 0.2388	-0.3783 to 0.2769	0.01277 to 0.6013	-0.2129 to 0.4351

p value	0.5721	0.742	**0.0425**	0.4701
			**∗**	

**UC**	OSDI vs. tBUT (s)	OSDI vs. Schirmer test (mm/5 min)	OSDI vs. Lissamine Green score	OSDI vs. LIPCOF

r (Pearson)	-0.5512	-0.3646	0.6049	0.607

95% CI	-0.7771 to -0.1995	-0.6589 to 0.02654	0.2841 to 0.8039	0.2871 to 0.8051

p value	**0.0043**	0.0671	**0.0011**	**0.001**
	**∗** **∗**		**∗** **∗**	**∗** **∗**

## Data Availability

The data that support the findings of this study are available on request from the corresponding author [Zsolt Barta]. The data are not publicly available due to state restrictions.
